# Multi-region assessment of pharmaceutical exposures and predicted effects in USA wadeable urban-gradient streams

**DOI:** 10.1371/journal.pone.0228214

**Published:** 2020-01-30

**Authors:** Paul M. Bradley, Celeste A. Journey, Daniel T. Button, Daren M. Carlisle, Bradley J. Huffman, Sharon L. Qi, Kristin M. Romanok, Peter C. Van Metre

**Affiliations:** 1 U.S. Geological Survey, Columbia, South Carolina, United States of America; 2 U.S. Geological Survey, Columbus, Ohio, United States of America; 3 U.S. Geological Survey, Lawrence, Kansas, United States of America; 4 U.S. Geological Survey, Beaverton, Oregon, United States of America; 5 U.S. Geological Survey, Lawrenceville, New Jersey, United States of America; 6 U.S. Geological Survey, Austin, Texas, United States of America; Universitat de Barcelona, SPAIN

## Abstract

Human-use pharmaceuticals in urban streams link aquatic-ecosystem health to human health. Pharmaceutical mixtures have been widely reported in larger streams due to historical emphasis on wastewater-treatment plant (WWTP) sources, with limited investigation of pharmaceutical exposures and potential effects in smaller headwater streams. In 2014–2017, the United States Geological Survey measured 111 pharmaceutical compounds in 308 headwater streams (261 urban-gradient sites sampled 3–5 times, 47 putative low-impact sites sampled once) in 4 regions across the US. Simultaneous exposures to multiple pharmaceutical compounds (pharmaceutical mixtures) were observed in 91% of streams (248 urban-gradient, 32 low-impact), with 88 analytes detected across all sites and cumulative maximum concentrations up to 36,142 ng/L per site. Cumulative detections and concentrations correlated to urban land use and presence/absence of permitted WWTP discharges, but pharmaceutical mixtures also were common in the 75% of sampled streams without WWTP. Cumulative exposure-activity ratios (EAR) indicated widespread transient exposures with high probability of molecular effects to vertebrates. Considering the potential individual and interactive effects of the detected pharmaceuticals and the recognized analytical underestimation of the pharmaceutical-contaminant (unassessed parent compounds, metabolites, degradates) space, these results demonstrate a nation-wide environmental concern and the need for watershed-scale mitigation of in-stream pharmaceutical contamination.

## Introduction

Human-use-pharmaceuticals in surface-water systems directly link human health with surface-water food-web structure and function (aquatic-ecosystem health) [1–8]. Pharmaceutical contaminants are intrinsic concerns in urban surface waters due to multiple wastewater point and non-point sources [2, 7, 9], aqueous mobility [10, 11], pH-variable activity [12], occurrence as complex mixtures [13], and designed biological activity [10, 11]; the latter often targeted at highly-conserved biological endpoints [5, 14–18], increasing the potential for adverse outcomes in multiple non-target organisms [1–4, 6, 18–24].

Wastewater treatment plants (WWTP) are generally cited as primary sources of pharmaceuticals to stream environments, in the United States (US) [7, 25] and globally [2, 10, 18, 26, 27]. Because WWTP surface-water outfalls are typically positioned to maximize dilution of effluent, most investigations of pharmaceutical occurrence and potential effects in stream ecosystems have focused on higher-order stream reaches [2, 10, 26, 27], despite the fact that headwater streams are fluvial capillaries [28] that dominate total stream length [29–33] and landscape-scale hydrologic connectivity [29–33] and provide critical habitat variability [30, 34, 35]. The US Geological Survey (USGS) National Water Quality Assessment (NAWQA) investigated pharmaceutical-contaminant concentrations in 59 wadeable headwater streams within the Piedmont ecoregion southern “megalopolis” [36, 37] during 2014 (Southeastern Stream Quality Assessment, SESQA [7, 38, 39]). The results illustrated that, while WWTP were indeed important point sources, extensive and diverse pharmaceutical-contaminant mixtures also were widely observed in urban stream settings with no National Pollution Discharge Elimination System (NPDES) permitted WWTP discharges, demonstrating the importance of non-WWTP sources and broad-scale mitigation approaches [7]. Notable SESQA results, such as the near-ubiquity of the anti-diabetic metformin, highlighted the fundamental biochemical link between global human-health crises, like Type II diabetes, and aquatic-ecosystem health and argued for assessment of headwater stream pharmaceutical exposures and potential effects in other regions across the US [7].

Herein, we expand the assessment of stream-ecosystem pharmaceutical risk (exposure and hazard) [40–42] to four US regions (including SESQA) sampled during 2014–2017 to test the hypothesis that pharmaceutical contamination is common across the US in urban-gradient headwater streams, including in those with no NPDES-permitted WWTP discharges. The potential for cumulative-contaminant effects (hazard) to in-stream biota was assessed based on 1) occurrence and cumulative concentrations of pharmaceutical mixtures, and 2) cumulative Exposure Activity Ratios (∑_EAR_) [43–45] based on high-throughput screening data in Toxicity Forecaster [ToxCast^™^, 46], as described in [44].

## Material and methods

### Site description and analytical method

**Fig 1:**
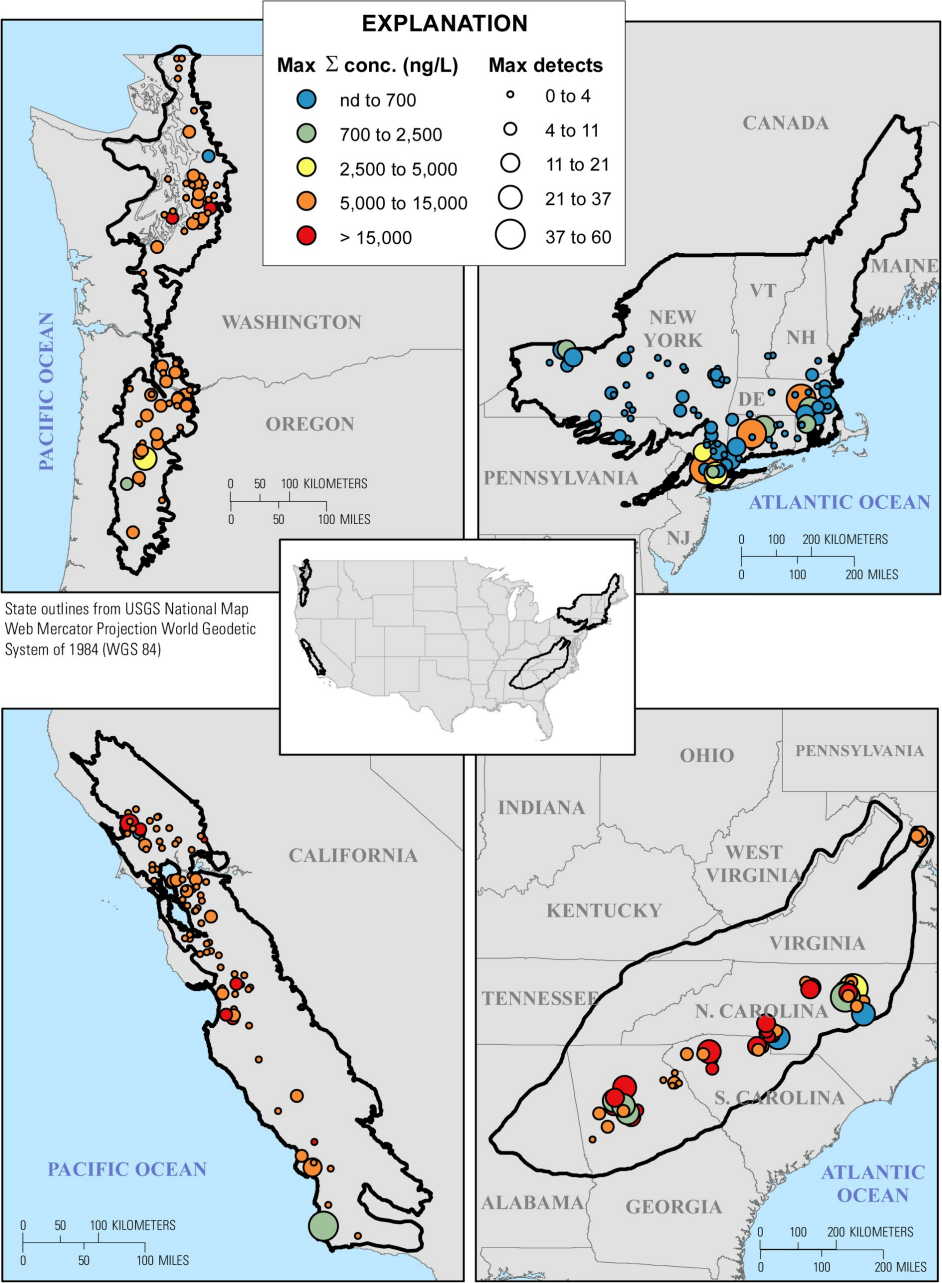
Cumulative maximum concentrations (ng/L) and numbers of pharmaceuticals detected at least once during the 2014–2017 synoptic samplings of water from wadeable streams in the Pacific Northwest (PNSQA, 2015; upper left), Northeast (NESQA, 2016; upper right), California (CaSQA, 2017; lower left), and Southeast (SESQA, 2014; lower right) regions as part of the USGS Regional Stream Quality Assessment (RSQA). For site details see S1 and S6 Tables. North is at top. Base-map image is from the USGS National Map [55].

Filtered water samples (10 mL) were collected by the USGS NAWQA Regional Stream Quality Assessments (RSQA) from perennial, wadeable (less than 10 m width and 1 m depth at base-flow) headwater stream sites in watersheds with varying degrees of urban and agricultural land use as part of four regional assessments (Pacific Northwest, PNSQA [47, 48]; California, CaSQA [49]; Northeast, NESQA [50, 51]; and SESQA [38, 39]) (Fig 1, S1 Table). During each regional water-quality assessment period (spring to summer), 3–5 water samples were collected from sites representing region-specific gradients in urban and agricultural development and a limited number of single samples were collected from nominal low-impact (low-development) watershed sites. Site selection and sampling methodologies are as described [38, 48, 50]. Samples were syringe filtered (0.7 μm pore size glass-fiber) into baked (500 C) amber glass vials and shipped on ice for analysis at the USGS National Water Quality Laboratory (NWQL) in Denver CO. Direct aqueous injection (100 μL), isotope dilution, high performance liquid chromatography tandem mass spectrometry (HPLC-MS/MS) was used to quantify an environmentally-relevant, representative subset consisting of 111 human-use pharmaceutical and pharmaceutical degradate compounds [52]; among these, gabapentin, guanylurea, and hexamethylenetetramine were added to the method in 2017 and analyzed only in CaSQA samples. Typically interpreted as a pesticide environmental contaminant, piperonyl butoxide also is medically indicated for treatment of lice [53, 54] and, thus, retained herein. Two additional analytes (atrazine, herbicide; methyl-1H-benzotriazole, solvent/deicing agent) included in the NWQL pharmaceutical method are not recognized pharmaceutical agents and are not included herein. Analytes, with Chemical Abstracts Services numbers and laboratory reporting limits (RL), are listed in the supporting information (S2 Table).

### Quality assurance quality control (QAQC)

HPLC-MS/MS pharmaceutical analysis included addition of 19 surrogate standards (400 ng/L nominal final concentration) to field-filtered samples to evaluate whole-method recovery (median = 102%, interquartile range [IQR] = 95–110%, range 2–320%). A total of 44 field blanks were prepared across all regions by processing pesticide-grade blank water through all field collection equipment and analyzing as above [52] for environmental samples. Only 9 of the 88 pharmaceutical analytes detected at least once in environmental samples in this study were also detected in field blanks. Among these, 6 (metformin, methadone, metoprolol, nevirapine, norethindrone, and omeprazole/esomeprazole) were only detected in a single field blank; corresponding environmental data were not blank adjusted, but interpretation of results below blank detection levels warrants caution. Lidocaine, nicotine, and caffeine were detected in 5 (11%), 5 (11%), and 2 (5%) field blanks, respectively; environmental concentrations less than corresponding 90^th^ percentile field blank concentrations were blank corrected to non-detect (nd).

### Data handling, statistics, and ∑_EAR_ analysis

The reporting limits for pharmaceutical analytes were determined using DQCALC^2^ software, a spreadsheet-based tool for graphically modeling relative standard deviation versus concentration and assigning a data precision statement in water analytical methods [56](RLDQC; S2 Table). Laboratory-estimated water concentrations below the RLDQC (positive detections with reduced quantitative certainty) were used as is (S3A–S5 Tables). Pharmaceutical results were aggregated to estimate maximum- and median-concentration exposures relative to the 108 pharmaceutical analytes (111 for CaSQA). S3A and S3B Table include the sample and detection counts, respectively, by compound and site for all pharmaceuticals detected at least once in the study. S4 Table includes the maximum concentrations of each pharmaceutical detected in this study by compound and site. S5 Table contains median concentrations (all samples) by compound and site and only includes those pharmaceuticals that were detected in at least half of the samples at one or more sites. S6 Table contains land-use/land-cover (LULC), specific conductivity, and major ion (maximum and median concentrations of Ca, Mg, Na, Cl, SO_4_, K) data.

As a first step, multivariate pharmaceutical detection or maximum/median concentration data matrices were log-transformed and normalized, converted to respective Euclidean-distance dissimilarity (similarity) matrices, and explored in three dimensions using non-metric multi-dimensional scaling (NMDS; Plymouth Routines in Multivariate Ecological Research, PRIMER v7; PRIMER-E Ltd., Plymouth, UK)[57]. Based on suggestive NDMS patterns, permutation-based one-way analysis of similarity (ANOSIM; permutation N = 999) was employed to test the null hypothesis (H_0_) of no difference between groups (i.e., that between-group dissimilarity was equivalent to or less than within-group dissimilarity), for *a priori* site groupings including study regions (CaSQA, NESQA, PNSQA, SESQA), urban centers (cities), and broad land-use categories (high urban, low urban, low-impact reference)(S7 Table). Cophenetic correlations between detection/concentration dissimilarity matrices and site-specific major ion or LULC Euclidean-distance dissimilarity matrices were assessed using the RELATE (H_0_: rho (ρ) = 0; permutation N = 999) routine (PRIMER v7)[57](S7 Table). Subsets of pharmaceutical, major ion, and LULC metrics that best explained the patterns in detection/concentration dissimilarity matrices were then identified using the permutation-based stepwise BEST(BVSTEP) (H_0_: ρ = 0; permutation N = 999) routine (PRIMER v7) with stop criteria of ρ > 0.95 or delta ρ < 0.001 (S7 Table). Finally, bivariate correlations (H_0_: rho (ρ) = 0; permutation N = 9999) between site-specific cumulative (sum of detected analytes) contaminant metrics (median detections and concentrations) and individual pharmaceutical, major ion, and LULC metrics identified above were assessed by nonparametric Spearman’s Rank-Order Correlation (Paleontological Statistics, PAST v3.25)(S8 and S9 Tables)[58]. The permuted (permutation N = 9999) probability that the centroids and dispersions of regional and WWTP-related site groupings were the same (H_0_: no difference between groups) was assessed using nonparametric One-way PERMANOVA on Euclidean distance [58–60].

Integrated effects of pharmaceutical contaminants were estimated, as described [44], using the toxEval package [61] of the open source statistical software R [62] to sum (presumptive additive effects [63–68]) individual EAR (ratio of detected concentration to activity concentration at cutoff (ACC) from Toxicity ForeCaster [ToxCast^™^; 46] high-throughput screening data [69, 70]) to provide site-specific cumulative EAR (∑_EAR_)[43–45, 71–73]. EAR ≥ 1 indicate exposures reported to modulate molecular targets in vitro, whereas EAR ≤ 1 suggest proportionately lower probability of biological activity. A recent comparison of EAR and benchmark-based Toxicity Quotients for select surface-water contaminants, for which both ToxCast and aquatic-toxicity benchmark data exist, indicated agreement between the commonly-employed 0.1 benchmark-based Toxicity Quotient threshold of concern and EAR = 0.001 [45]. Non-specific-endpoint, baseline, and unreliable response-curve assays were excluded [as described in 43, 44, 45, 71–73](S10 Table). ∑_EAR_ results are summarized in S11–S14 Tables.

No permits were required for this work. All data are also available from the USGS National Water Information System (NWIS) [74] and from USGS ScienceBase [75].

## Results and discussion

### Pharmaceutical mixtures were common in all regions

**Fig 2:**
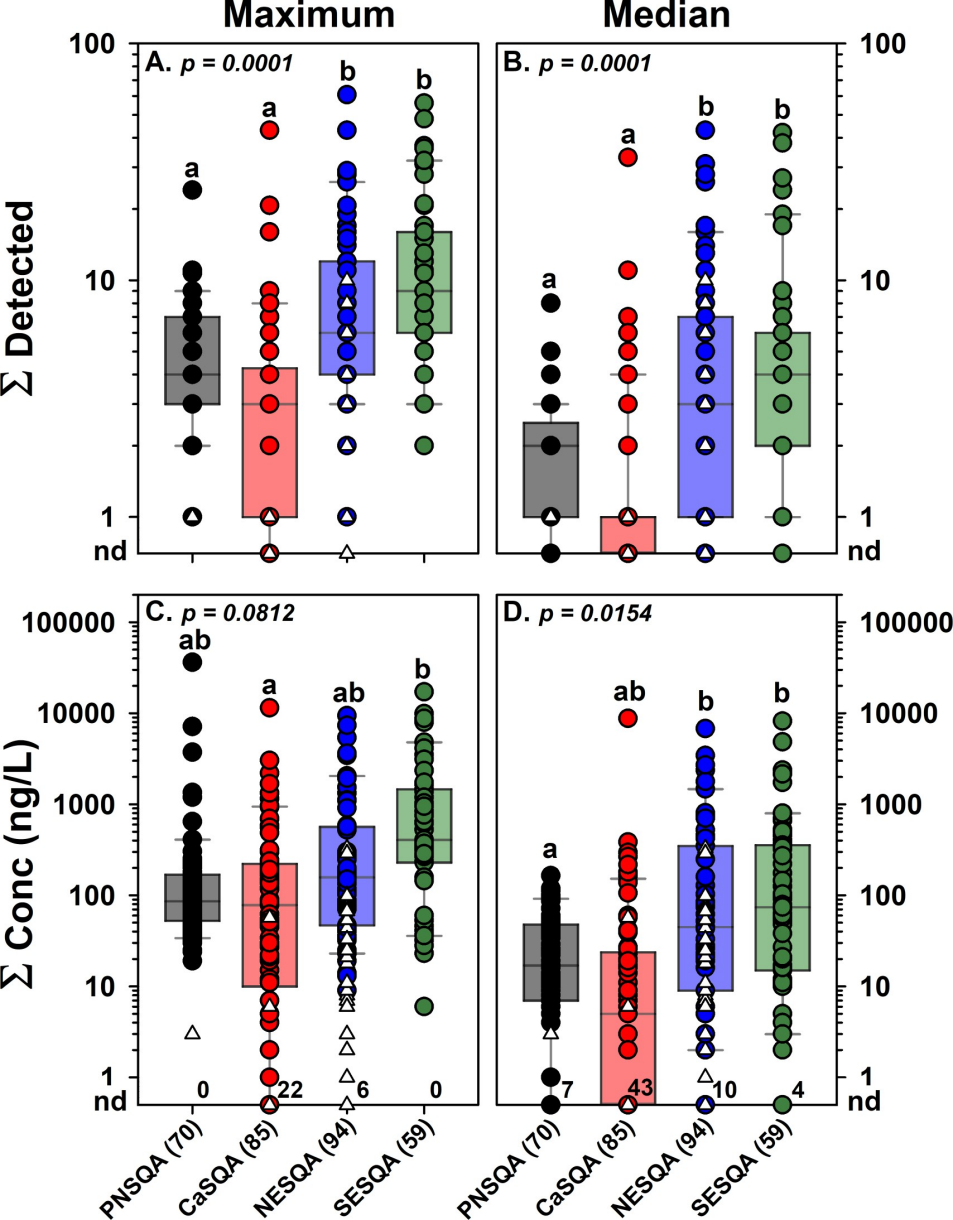
**Cumulative maximum (left) and median (right) concentrations (ng/L; bottom) and numbers of pharmaceuticals detected in water samples from wadeable streams in each region (total sites in parentheses) as part of the USGS Regional Stream Quality Assessment (RSQA).** Circles indicate maximum (left) or median (right) data for sites sampled multiple times (n = 3–5). Open triangles indicate data from single-sample sites and do not differ between maximum and median plots. Boxes, centerlines, and whiskers indicate interquartile range, median, and 5^th^ and 95^th^ percentiles, respectively, for multiple-sample sites (circles), only. For each plot, p is the permuted probability that the centroids and dispersions across all groups are the same and different letters indicate groups with pairwise probabilities that are less than 0.05 (PERMANOVA) for multi-sample sites only. Numbers above X-axes (bottom plots) indicate numbers of sites in each region with no detections (nd) under maximum and median exposure conditions.

Among the 308 wadeable streams sampled across all regions during the 2014–2017 RSQA studies, multiple pharmaceuticals (pharmaceutical mixtures) were detected at least once (maximum exposure dataset) in 95% (248) of the 261 multiple-sample, urban-gradient sites and in 68% (32) of the 47 single-sample, non-urban, presumptive low-impact, sites (Fig 1, S3B and S4 Tables). Importantly, pharmaceutical mixtures were detected in at least half of the samples (median exposure dataset) from 81% (212) of the multiple-sample, urban-gradient sites (S1 Fig, S5 Table). Cumulative (sum of detected pharmaceuticals) maximum and median detections ranged 0–60 per site (median: 4; IQR: 2–8) and 0–43 per site (median: 2; IQR: 1–4), respectively, with the centroid (mean) of cumulative pharmaceutical detections per site estimated (PERMANOVA) to be greater (permutation N = 9999 probability of being the same = 0.0001) in eastern (NESQA, SESQA) than in western (PNSQA, CaSQA) study streams (Fig 2A and 2B, S4 and S5 Tables). Site-specific cumulative maximum and median concentrations ranged nd-36,142 ng/L (median: 88 ng/L; IQR: 29–306) and nd-8,756 ng/L (median: 19 ng/L; IQR: 3–75 ng/L), respectively (Fig 2C and 2D, S4 and S5 Tables). The cumulative maximum concentrations were not clearly different (PERMANOVA; probability of being the same, p = 0.0812) between regions. There is some evidence to suggest the centroid of cumulative median concentrations was greater (permutation N = 9999 probability of being the same = 0.0154) in the eastern region (NESQA, SESQA) streams than in PNSQA streams, with CaSQA streams intermediate, but extensive overlap of the data distributions was apparent in all cases. The apparently greater detections and, possibly, median concentrations of pharmaceuticals between eastern and western regions are consistent with reported differences in cumulative 2016 prescription drug sales (87 vs 50 million US dollars [76]) and populations (79 vs 50 million people [77]) between corresponding eastern and western region states, respectively.

**Fig 3:**
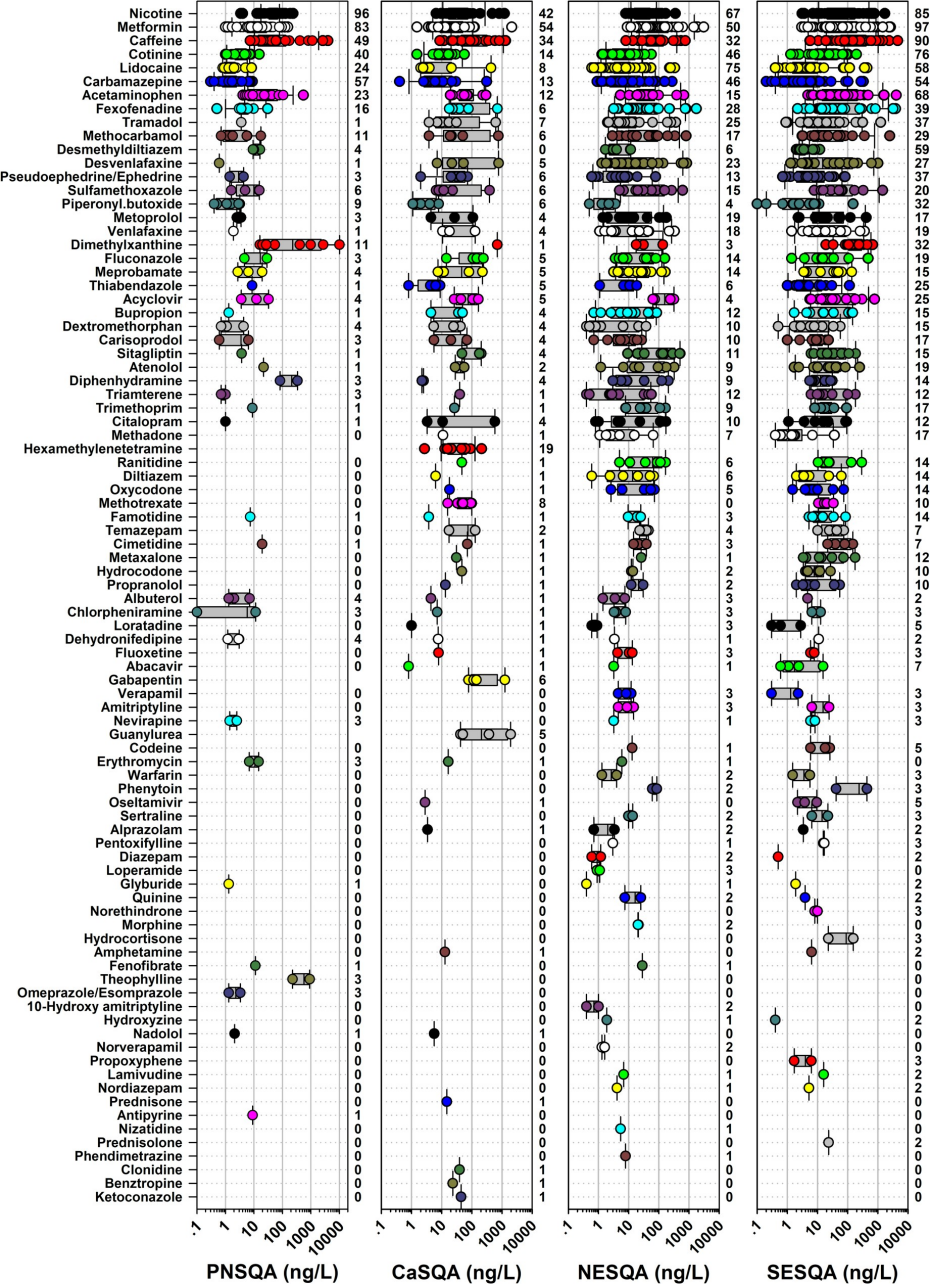
Maximum concentrations (ng/L) of 88 pharmaceuticals detected at least once (in order of decreasing number of detections across all regions and samples) in water samples from wadeable streams in each region as part of the USGS Regional Stream Quality Assessment (RSQA). Circles are data for individual sites. Boxes, centerlines, and whiskers indicate interquartile range, median, and 5^th^ and 95^th^ percentiles, respectively. Numbers to the right of each plot indicate the percentage of sites within the region at which the compound was detected at least once. Gabapentin, guanylurea, and hexamethylenetetramine were analyzed only in CaSQA samples.

Eighty-eight of the 108 pharmaceutical analytes (111 in CaSQA) were detected at least once (Fig 3). Nicotine and its metabolite, cotinine, were detected at 70% and 47% of sites, respectively. Consistent with the SESQA findings reported earlier [7], the type-II diabetes medicine, metformin, was common (68% of sites) across all sites and regions. Caffeine-related compounds, caffeine and 1,7-dimethylxanthine, were detected at least once at 42% and 10% of sites, respectively. Other frequently detected (detected at more than 50 sites or 15% of sites) compounds included lidocaine (intravenous/topical analgesic, 42%), carbamazepine (anti-seizure medication, 41%), acetaminophen (oral analgesic, 26%), fexofenadine (anti-histamine, 21%), and tramadol (opioid analgesic, 17%); These pharmaceuticals also exhibited the highest maximum and median concentrations observed in this study (Fig 3 and S2 Fig). The numbers of frequently detected (detected at more than 15% of sites in the region) pharmaceuticals were higher in eastern regions (NESQA: 14; SESQA: 29) than in western regions (PNSQA: 8; CaSQA: 3), consistent with previously noted regional differences in cumulative 2016 prescription drug sales [76] and population estimates [77].

Likewise, the spatial detection frequencies (the percentage of study sites at which analytes were detected) of several individual pharmaceuticals and pharmaceutical groups differed markedly between regions (Fig 3 and S2 Fig). For example, the antidepressants, venlafaxine and desvenlafaxine, were detected at a substantially higher (approximately four times higher) percentage of eastern region sites than western region sites. The percentages of eastern region study sites, at which pharmaceuticals associated with seasonal and perennial allergies (fexofenadine, pseudoephedrine/ephedrine, diphenhydramine, loratadine) were detected, were generally double those observed in the two western study regions (Fig 3), despite similar reported percentages of hay fever and respiratory allergies for children [78] and adults [79] in the eastern and western US in 2018. Correlations between co-detected analytes are presented in S8 Table.

### Land-use/land-cover and major ion predictors of pharmaceuticals

Frequent occurrence, multiple detections per site (median: 4 compounds per site across all sites including in pre-selected low-impact watersheds), and elevated cumulative concentrations (up to >36 μg/L per site) emphasize the need for identification, monitoring, and mitigation of pharmaceutical sources in wadeable-stream ecosystems. ANOSIM analysis of site-specific pharmaceutical and LULC data matrices indicated marginal (Global R range: 0.043–0.239) differences between RSQA study unit, urban-center, and ecoregion groupings (S7 Table). Likewise, weak correlations (RELATE; ρ range: 0.029–0.116) were found between median pharmaceutical similarity matrices (detection/concentrations under median conditions) and watershed LULC similarity matrices, with multiple individual urban development metrics (e.g., percentage of developed industrial/military [Dev_IndusMilitary2012] and semi-developed anthropogenic other [SemiDev_AnthroOth2012] land cover from the National Wall-to-Wall Anthropogenic Land Use Trends (NWALT) 1974–2012 database [80]; estimated road density [RoadDensity2016] from the 2016 U.S. Census data [81]) and wastewater metrics (e.g., number of NPDES-permitted wastewater facilities [NumFacilities2012] in 2012 [82]) consistently the strongest among the identified correlates (BEST routine; S7 Table).

Based on these suggestive multi-variate results, bivariate correlations between site-specific summary metrics (cumulative detections/concentrations) under the estimated median exposure conditions and readily available LULC metrics identified by BEST were further explored using Spearman Rank-Order Correlation (S9 Table). Several watershed urbanization metrics (e.g., percentage land cover as high-density urban development [DevelopedHigh2011] from the 2011 National Land Cover Database [83]) correlated well with cumulative detections (ρ range: 0.475–0.543) and concentrations (ρ range: 0.424–0.525) under median exposure conditions (S9, S15 and S16 Tables). Weaker correlations (ρ range: 0.294–0.395) were observed between various wastewater discharge metrics (e.g., number of NPDES-permitted wastewater facilities [NumFacilities2012] in 2012 [82]) and cumulative median pharmaceutical detections or concentrations (S9, S15 and S16 Tables). Differences (permutation N = 9999 probability of being the same = 0.0001) in the centroids of cumulative median detections and concentrations were observed between sites with (detections median: 3, IQR: 2–9; cumulative concentrations median: 62 ng/L; IQR: 20–405 ng/L) and without permitted wastewater discharges (detections median: 1, IQR: 0–3; cumulative concentrations median: 11 ng/L; IQR: 0–55 ng/L). These results are consistent with the documented importance of WWTP discharges as pharmaceutical-contaminant sources [84, 85] and the substantial pharmaceutical-contaminant reductions in urban-area wadeable streams following WWTP-treatment upgrades [84, 85] or WWTP closures [86, 87].

The pharmaceutical contaminants observed at the 75% of RSQA sites without NPDES-permitted discharges, however, confirm previous conclusions that WWTP outfalls are not the only important pathways of pharmaceutical contaminants to urban/suburban streams [1, 7, 88, 89]. Other potential urban-gradient sources of pharmaceuticals to streams include aging sewer infrastructure [90, 91], combined (sanitary/stormwater) sewer overflows [92–96], private septic and on-site waste-handling systems [97–99], gray-water systems [100–102], green space and golf course wastewater reuse [103], and animal waste runoff [104–106]. Notably, a recent national reconnaissance demonstrated that untreated stormwater can be an important episodic source of mixed pharmaceuticals to surface waters, at levels comparable to and often exceeding those in treated WWTP effluent [107]. Thus, these results reiterate the need for whole-watershed, contaminant-mitigation approaches, including improved pharmaceutical disposal practices, wastewater treatment and transfer systems, and stormwater controls.

More, the results argue for research and implementation of new high-frequency or continuous sensor technologies for direct or indirect monitoring of pharmaceutical contaminants in next generation water observing systems [e.g., 108, 109] in urban settings. Surface-water ions (and related conductivity measures) have been suggested as potentially useful surrogates for indirect monitoring of pharmaceutical contamination in streams [e.g., 110, 111], because physiological ions (electrolytes) and pharmaceuticals are both primarily excreted in urine [112–115] and are frequently reported together in wastewater-impacted streams at locally-elevated concentrations [e.g., 92, 93]. The most useful monitoring approaches are expected to be fixed-station or single watershed applications [111], due to the potential confounding effects of site-to-site variability in non-wastewater sources of ions in urban-gradient streams [116, 117], including geologic minerals [118], fertilizer runoff [104], road salt [119, 120], and concrete infrastructure [121–123]. Nevertheless, the current multi-region dataset provides a unique opportunity to test the broad geospatial validity of the approach by assessing the probability (p) of no correlation (H_0_: rho (ρ) = 0; permutation N = 9999; Spearman Rank-Order Correlation) between in-stream pharmaceutical and ion concentrations (S9 Table).

The results support the potential utility of surface-water ions as surrogates for wastewater-associated contaminants like pharmaceuticals [110, 111]. Although no relation (p ≥ 0.2528) was observed between specific conductivity and cumulative pharmaceutical metrics under median exposure conditions, correlations were observed between pharmaceuticals and sodium or chloride (p ≤ 0.0182), with the strongest correlations observed for potassium (p ≤ 0.0001). Comparison of the Spearman correlation coefficients provided additional insight into the importance of the yellow-water/wastewater pathway relative to potential confounders (i.e., non-wastewater sources [104, 116–123]). Correlations between median pharmaceutical metrics and median concentrations of sodium and chloride were weak (ρ ≤ 0.202), consistent with numerous confounding non-wastewater sources of these ions in urban settings [116, 119, 120]. However, promising correlations (ρ range: 0.314–0.328) were observed between median concentrations of potassium and cumulative median detections and concentrations of pharmaceuticals, indicating the potential for potassium as an indicator of in-stream pharmaceutical contamination in fixed-place or single watershed applications and consistent with the strong correlation (R^2^ > 0.89) reported between instream concentrations of potassium and pharmaceuticals in the Leine River watershed in Germany [111]. The stronger broad regional correlation in this study between potassium and pharmaceuticals may reflect comparatively less variability in non-wastewater potassium sources as well as the usage of potassium salts (e.g., ferrate, ferrocyanate) as floculants/coagulants in wastewater treatment [124]. Emerging sensor technologies that hold promise for next generation monitoring of potassium (and other ions) include recently described nanorod-based potassium ion sensors [125] and multi-parameter potentiometric microanalyzers (lab-on-a-chip platforms) developed for space travel [126–128] and environmental water quality monitoring [129, 130].

### Potential for mixed-pharmaceutical biological effects

The 111-pharmaceutical analytical space assessed in this study is a fractional indicator of the presumptive pharmaceutical-contaminant universe, with more than 4000 active ingredients (parent compounds) [2, 131] in current use and an unknown chemical-space [132] of metabolites and environmental degradates [13]. Given the breadth of species, life stages, biomasses, and concomitant vulnerabilities present in urban-gradient aquatic food webs [133–136] and the designed bioactivity of commercial pharmaceuticals [1–6, 14–17, 19–24, 137], their detection in RSQA headwater streams is *prima facie* evidence of the potential for molecular toxicity and sub-lethal effects in non-target, organisms in urban-gradient headwater streams across the US [138–141].

**Fig 4:**
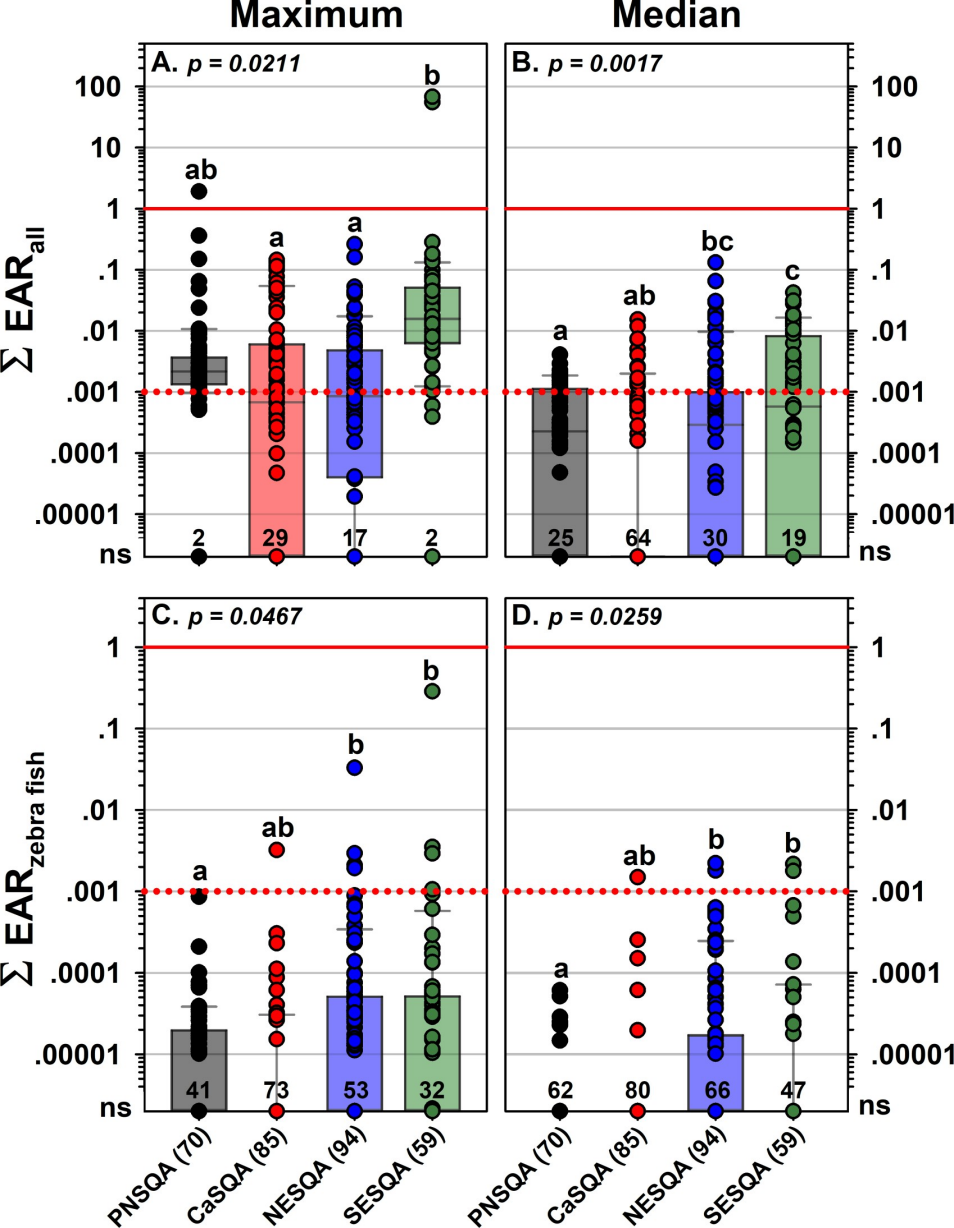
**Cumulative maximum (left) and median (right) Exposure-Activity Ratios (Σ**_**EAR**_**) of ToxCast all (primarily molecular, top) and zebra-fish only (bottom) endpoints for pharmaceuticals detected in water samples from wadeable streams in each region (total sites in parentheses) as part of the USGS Regional Stream Quality Assessment (RSQA**). Boxes, centerlines, and whiskers indicate interquartile range, median, and 5^th^ and 95^th^ percentiles, respectively, for multiple-sample sites (circles), only. For each plot, p is the permuted probability that the centroids and dispersions across all groups are the same and different letters indicate groups with pairwise probabilities that are less than 0.05 (PERMANOVA) for multi-sample sites only. Numbers above X-axes in each plot indicate number of sites with no significant activity (Σ_EAR_ < 0.00001).

The *in vitro* ToxCast-based EAR approach provides an additional line of evidence for sub-lethal effects at a reported concentration [43, 73], supports estimation of cumulative effects (∑_EAR_) of mixed-contaminant exposures using the CA-model methodology [63–68], and predicts probable effects consistent with traditional *in vivo* water-quality benchmark-based toxicity quotient (TQ) approaches (EAR = 0.001 comparable to commonly-employed TQ = 0.1 effects threshold) [45]. ToxCast [46] includes exposure-response metrics for 9000+ organic chemicals and approximately 1000 standardized, predominantly-vertebrate, molecular bioassay endpoints (e.g., nuclear receptor, DNA binding) [70, 142, 143]. ToxCast EAR results for estimated maximum and median pharmaceutical exposure conditions are summarized in Fig 4 and S11–S14 Tables. Given the diversity of organisms and concomitant range of vulnerabilities in surface-water food webs [133–136], we employed the recently suggested effects-screening threshold of 0.001 [45], as described [44]. Of the 88 pharmaceuticals detected at least once in this study, only 43% (38) had acceptable ToxCast data at the time of access. Under maximum exposure conditions, 63% (194) of study sites had one or more compounds with individual EAR greater than the 0.001 effects-screening threshold, 65% (201) had cumulative EAR (ΣEAR_max_) ≥ 0.001, and 3 sites had ΣEAR_max_ ≥ 1 (Fig 4). These results indicate transient exposures with a probability of vertebrate molecular effects were common in urban-gradient headwater streams across the US. Of the 62 pharmaceuticals in the estimated median exposure dataset, 61% (38) had exposure-effects data in ToxCast. Approximately 25% of the study sites had individual or cumulative EAR_med_ ≥ 0.001 under the estimated median exposure conditions (Fig 4), indicating that sites with persistent exposures with a probability of molecular effects were common in urban headwater streams across the US. Zebra fish (ZF; *Danio rario*) embryo metrics in ToxCast inform organism-level as well as vulnerable, early-life-cycle effects in fish [144, 145]. Thus, the results also indicate the potential for pharmaceutical effects to fish at the organism level in at least some headwater streams, because 7 and 5 sites, respectively, had ΣEAR_max ZF_ and ΣEAR_med ZF_ ≥ 0.001 across all ZF endpoints.

### Implications for stream ecosystem health and remediation

The results indicate substantial pharmaceutical-contaminant concerns in wadeable, urban-gradient, headwater streams not only in SESQA [7], but in other regions across the US [1, 4, 6, 19], irrespective of WWTP discharge. Crucially, the pharmaceutical-analyte space [52] assessed herein is an order(s) of magnitude underestimate of the presumptive pharmaceutical-contaminant universe, with 4000+ parent compounds in current use [2] and unquantified numbers of environmental metabolites/degradates [13, 132]. Considering only those pharmaceuticals assessed in this study, individual concentrations up to μg/L levels and multiple detections per site (median = 4 across all sites including pre-selected, low-impact watersheds) at cumulative concentrations ranging more than 36 μg/L are notable concerns, given documented adverse impacts of individual pharmaceuticals at low ng/L concentrations [20] and the widespread cumulative exceedances of *in vitro* molecular effects thresholds (∑_EAR_) observed in these urban-impacted headwater streams.

Notably, these pharmaceutical results confirm earlier concerns about the linkage between human- and aquatic-health [7, 27, 146, 147]. Echoing earlier SESQA-only results [7], the Type II diabetes medicine, metformin again was the most consistently detected pharmaceutical across all sites and samples (more than half of the samples at 57% of sites; detected at least once at 68% of sites) at site-specific maximum concentrations ranging nd-3,077 ng/L (median: 11 ng/L; IQR: nd-46 ng/L), despite the fact that NPDES discharges occurred in only 25% of the study watersheds. In 2017 when the metabolite, guanylurea, was added to the analytical portfolio, metformin and guanylurea were detected at least once in 54% (46/85) and 5% (4/85) of CaSQA sites, respectively. The anti-diabetic pharmaceuticals, sitagliptin and glyburide, also were detected in 8% (23/308) and 1% (3/308) of the sites in this study, respectively. Importantly, a number of other potentially diabetes-related pharmaceuticals were widely detected in this study, including several analgesics (e.g., tramadol [17% of sites], desvenlafaxine [14%], venlafaxine [10%], gabapentin [2%], amitriptyline [2%]), prescribed for treatment of peripheral neuropathic pain, a common symptom of progressive Type II diabetes [148–152].

Metformin was the second most frequently detected pharmaceutical across all sites in this study and the fourth most prescribed pharmaceutical in the US, with an estimated 81 million prescriptions in 2016 alone [153, 154]. US and global metformin usage is expected to increase, as a first-line diabetes therapy [155–157] and treatment candidate for polycystic ovarian syndrome [158] and cancers [159]. Metformin is excreted essentially unchanged in human urine [160], poorly removed by wastewater treatment technologies [161], considered environmentally recalcitrant [161, 162], and increasingly reported in environmental samples [7, 9, 163]. Environmentally-relevant [161, 164, 165] metformin exposures in the μg/L range have recently been shown to induce biological responses in fish [166–169], including up-regulation of vitellogenin mRNA [170, 171] and other gene targets [169, 171, 172], male intersex in fathead minnow (*Pimephales*) [170], and behavioral modifications in Siamese fighting fish (*Betta splendens*) [167]. Guanylurea, metformin’s only currently recognized persistent environmental degradate, is often observed in surface waters at higher concentrations than metformin [155, 163–165] and, importantly, has been recently reported to cause growth effects in Japanese medaka (*Oryzias latipes*) similar to metformin but at low (<10) ng/L concentrations [166].

Fish and fish-embryos are widely-used animal models in the pharmaceutical development pipeline [173, 174], including for anti-diabetics [175, 176]; from this perspective, fish are arguably pharmaceutical target organisms with unintended environmental exposures. Consistent with this use, individual and simple mixtures of pharmaceuticals have been shown to cause unintended effects to the health and behavior of laboratory and wild fish at environmentally-relevant concentrations [24, 177–179] and the potential biological impacts of characteristically complex environmental pharmaceutical cocktails are global concerns [64, 68, 180, 181]. In light of the documented potential for pharmaceutical bioconcentration [182–184] and trophic transfer [182] within aquatic food webs and for trophic transfer of pharmaceuticals from aquatic to riparian food webs [185], measured water concentrations may substantially underestimate the ecological exposures and effects from in-stream pharmaceutical contaminants [182, 185]. Thus, considering potential individual and interactive effects of the 88 pharmaceuticals detected in headwater streams herein and the recognized orders-of-magnitude analytical underestimation of the presumptive pharmaceutical-contaminant (parent compounds, metabolites, degradates) space, the results of the present study demonstrate a nation-wide need for watershed-scale pharmaceutical-contaminant mitigation approaches that extend the current emphasis on WWTP-effluent sources to include more broadly distributed inputs such as septic systems, leaking wastewater transfer systems, and urban stormwater runoff.

## Supporting information

S1 Fig Cumulative median concentrations and detections of pharmaceuticals (ng/L) detected in at least half of the water samples at one or more wadeable stream sites during the 2014–2017 synoptic samplings in the Pacific Northwest (PNSQA, 2015; upper left), Northeast (NESQA, 2016; upper right), California (CaSQA, 2017; lower left), and Southeast (SESQA, 2014; lower right) regions as part of the USGS Regional Stream Quality Assessment (RSQA). For site details see S1 and S6 Tables. North is at top. Base-map image is from the USGS National Map [55].
(XLSX)

S2 Fig Median concentrations ng/L of pharmaceuticals detected in at least half of the water samples (in order of decreasing number of detections across all regions and samples) at one or more wadeable stream sites in the (left to right) Pacific Northwest (PNSQA, 2015), Northeast (NESQA, 2016), California (CaSQA, 2017), and Southeast (SESQA, 2014) regions as part of the USGS Regional Stream Quality Assessment (RSQA). Circles are data for individual sites. Boxes, centerlines, and whiskers indicate interquartile range, median, and 5th and 95th percentiles, respectively. Numbers to the right of each plot indicate the percentage of sites at which the compound was detected at least once. Gabapentin, guanylurea, and hexamethylenetetramine were analyzed only in CaSQA samples.
(XLSX)

S3 Fig Spearman rank-order correlation plot for cumulative median concentrations and detections as well as median individual concentrations of pharmaceuticals (ng/L) detected in at least half of the water samples at one or more wadeable stream sites during the 2014–2017 synoptic samplings in the Pacific Northwest (PNSQA, 2015), Northeast (NESQA, 2016), California (CaSQA, 2017), and Southeast (SESQA, 2014) regions as part of the USGS Regional Stream Quality Assessment (RSQA). (XLSX)

S4 Fig Spearman rank-order correlation plot for cumulative median concentrations and detections of pharmaceuticals (ng/L), median concentrations of major ions, and GIS LULC metrics at one wadeable stream sites during the 2014–2017 synoptic samplings in the Pacific Northwest (PNSQA, 2015; upper left), Northeast (NESQA, 2016; upper right), California (CaSQA, 2017; lower left), and Southeast (SESQA, 2014; lower right) regions as part of the USGS Regional Stream Quality Assessment (RSQA). (XLSX)

S1 Table Site information and select summary analytical results (detections and concentrations in nanograms per liter, ng/L) for wadeable streams sampled during 2014–2017 as part of the USGS Regional Stream Quality Assessment (RSQA) synoptic samplings in the Pacific Northwest (PNSQA, 2015), Northeast (NESQA, 2016), California (CaSQA, 2017), and Southeast (SESQA, 2014) regions. (XLSX)

S2 Table Pharamceutical compound information for analyses performed by the USGS National Water Quality Laboratory (NWQL) as part of the USGS Regional Stream Quality Assessment (RSQA) synoptic samplings in the Pacific Northwest (PNSQA, 2015), Northeast (NESQA, 2016), California (CaSQA, 2017), and Southeast (SESQA, 2014) regions. Gabapentin, guanylurea, and hexamethylenetetramine were analyzed only in CaSQA samples.
(XLSX)

S3 Table a. Number of pharmaceutical water samples collected during USGS Regional Stream Quality Assessment (RSQA) synoptic samplings in the Pacific Northwest (PNSQA, 2015), Northeast (NESQA, 2016), California (CaSQA, 2017), and Southeast (SESQA, 2014) regions. b. Number of detections for pharmaceuticals detected at least once during the USGS Regional Stream Quality Assessment (RSQA) synoptic samplings in the Pacific Northwest (PNSQA, 2015), Northeast (NESQA, 2016), California (CaSQA, 2017), and Southeast (SESQA, 2014) regions.
(XLSX)

S4 Table Maximum detected concentration (nanograms per liter, ng/L) of pharmaceuticals detected at least once during the USGS Regional Stream Quality Assessment (RSQA) synoptic samplings in the Pacific Northwest (PNSQA, 2015), Northeast (NESQA, 2016), California (CaSQA, 2017), and Southeast (SESQA, 2014) regions. (XLSX)

S5 Table Median detected concentration (nanograms per liter, ng/L) of pharmaceuticals detected in at least half of the water samples during the USGS Regional Stream Quality Assessment (RSQA) synoptic samplings in the Pacific Northwest (PNSQA, 2015), Northeast (NESQA, 2016), California (CaSQA, 2017), and Southeast (SESQA, 2014) regions. (XLSX)

S6 Table Watershed-specific GIS metrics (see S15 Table for GIS data dictionary) and median and maximum major ion concentrations (milligrams per liter, mg L-1) for wadeable streams in the USGS Regional Stream Quality Assessment (RSQA) synoptic samplings in the Pacific Northwest (PNSQA, 2015), Northeast (NESQA, 2016), California (CaSQA, 2017), and Southeast (SESQA, 2014) regions. (XLSX)

S7 Table Summary statistics for multivariate relations between pharmaceutical cumulative detection/concentration (log-transformed and normalized) data matrices (Euclidean distance) and site-specific LULC or major ion data matrices (Euclidean distance) assessed by non-metric multi-dimensional scaling (NMDS), one-way analysis of similarity (ANOSIM), and permutation-based (permutations = 999) cophenetic correlation (RELATE) routines. (XLSX)

S8 Table Spearman rho (r) rank-order correlation coefficients (r; lower triangle) and 2-tail probability (permutation N = 9999) that no correlation exists (upper triangle) for site-specific cumulative pharmaceutical detection and concentrations and individual pharmaceutical concentrations (nanograms per liter, ng/L) under estimated maximum (max) or median (med) exposure conditions. (XLSX)

S9 Table Spearman rho (r) rank-order correlation coefficients (r; lower triangle) and 2-tail probability (permutation N = 9999) that no correlation exists (upper triangle) for site-specific cumulative median pharmaceutical detections or concentrations (nanograms per liter, ng/L), major ions, and select GIS metrics identified by multi-variate RELATE and BEST analyses. (XLSX)

S10 Table Compound:Endpoint combinations excluded from ToxCast evaluation due to unreliable concentration-response relationship and resulting lack of confidence in activity concentration at cutoff (ACC). (XLSX)

S11 Table Site-specific Exposure Activity Ratios (EAR) under Maximum exposure conditions for those compounds with exact Chemical Abstract Service (CAS) number matches and with reliable concentration-response relationship and ACC data in ToxCast. (XLSX)

S12 Table Site-specific Exposure Activity Ratios (EAR) under Maximum exposure conditions for all bioassay endpoints within each class shown. Data are for those compounds with exact Chemical Abstract Service (CAS) number matches and with reliable concentration-response relationship and ACC data in ToxCast.
(XLSX)

S13 Table Site-specific Exposure Activity Ratios (EAR) under Median exposure conditions for those compounds with exact Chemical Abstract Service (CAS) number matches and with reliable concentration-response relationship and ACC data in ToxCast. (XLSX)

S14 Table Site-specific Exposure Activity Ratios (EAR) under Median exposure conditions for all bioassay endpoints within each class shown. Data are for those compounds with exact Chemical Abstract Service (CAS) number matches and with reliable concentration-response relationship and ACC data in ToxCast.
(XLSX)

S15 Table Data dictionary describing metrics in S9 Table. Additional citation details provided in S16 Table.
(XLSX)

S16 Table Additional citation information for metrics in S9 and S15 Tables. (XLSX)
